# Identification and characterization of intervening sequences within 23S rRNA genes from more than 200 *Campylobacter *isolates from seven species including atypical campylobacters

**DOI:** 10.1186/1471-2180-9-256

**Published:** 2009-12-11

**Authors:** Akihiro Tazumi, Yuki Kakinuma, Naoaki Misawa, John E Moore, Beverley C Millar, Motoo Matsuda

**Affiliations:** 1 Laboratory of Molecular Biology, School of Environmental Health Sciences, Azabu University, Sagamihara 229-8501, Japan; 2 Department of Veterinary Public Health, Faculty of Agriculture, University of Miyazaki, Miyazaki 889-2192, Japan; 3 School of Biomedical Sciences, University of Ulster, Cromore Road, Coleraine, Co Londonderry, BT52 1SA, Northern Ireland, UK; 4 Department of Bacteriology, Northern Ireland Public Health Laboratory, Belfast City Hospital, Belfast, BT9 7AD, Northern Ireland, UK

## Abstract

**Background:**

Identification and characterization of intervening sequences (IVSs) within 23S rRNA genes from *Campylobacter *organisms including atypical campylobacters were carried out using two PCR primer pairs, designed to generate helix 25 and 45 regions.

**Results:**

Only *C. sputorum *biovar sputorum LMG7975 and fecalis LMG8531, LMG8534 and LMG6728 of a total of 204 *Campylobacter *isolates (n = 56 *C. jejuni*; n = 11 *C. coli*; n = 33 *C. fetus*; n = 43 *C. upsaliensis*; n = 30 *C. hyointestinalis*; n = 4 *C. sputorum *biovar sputorum; n = 5 *C. sputorum *biovar fecalis; n = 5 *C. sputorum *biovar paraureolyticus; n = 10 *C. concisus*; n = 7 *C. curvus*) were shown to carry IVSs in helix 25 region. *C. sputorum *biovar fecalis LMG8531 and LMG8534, interestingly, carried two different kinds of the 23S rRNA genes with and without the IVS, respectively. Consequently, in a total of 265 isolates of 269, including 65 *C. lari *isolates examined previously, the absence of IVSs was identified in the helix 25 region. In the helix 45 region, all the *C. hyointestinalis*, *C. sputorum *and *C. concisus *isolates were shown not to carry any IVSs. However, the 30 of 56 *C. jejuni *isolates (54%), 5 of 11 *C. coli *(45%), 25 of 33 *C. fetus *(76%), 30 of 43 *C. upsaliensis *(70%) and 6 of 7 *C. curvus *(90%) were shown to carry IVSs. In *C. jejuni *and *C. upsaliensis *isolates, two different kinds of the 23S rRNA genes were also identified to occur with and without IVSs in the helix 45 region, respectively.

**Conclusions:**

Secondary structure models were also constructed with all the IVSs identified in the present study. In the purified RNA fractions from the isolates which carried the 16S or 23S rRNA genes with the IVSs, no 16S or 23S rRNA was evident, respectively.

## Background

Thermophilic *Campylobacter *species, primarily *Campylobacter jejuni *and *C. coli *are the most frequently recognized cause of acute bacterial gastroenteritis in humans in the Western world. In relation to human campylobacteriosis, *C. upsaliensis*, *C. hyointestinalis*, *C. lari*, *C. fetus *and *C. sputorum *biovar sputorum have also been demonstrated to be implicated as gastrointestinal pathogens though these are rare [1,2]. These *Campylobacter *organisms have also been isolated from animals.

Moreover, *C. concisus*, *C. curvus *and so on are detected in association with the oral cavity [3]. Alternatively, *C. sputorum *biovar fecalis is isolated from animals [4]. A multiplex PCR assay has recently developed for the identification of *C. coli*, *C. fetus*, *C. hyointestinalis *subsp. hyointestinalis, *C. jejuni*, *C. lari *and *C. upsaliensis *[5]. Thus, at this time, the genus *Campylobacter *comprises 18 species [6]

As already shown, the genus *Campylobacter *is, in general, indicated to carry the three copies of rRNA gene operon [7-9]

In relation to bacterial 23S rRNA genes, the occurrence of intervening sequences (IVSs) [10-12] and the fragmentation of 23S rRNA [13-16] have been demonstrated.

In the genus *Campylobacter*, the ε-subdivision of the *Proteobacteria*, the occurrence of internal transcribed spacers was first described in helix 45 region within 23S rRNA gene in two of four *C. jejuni*, in both *C. fetus *and in one of two *C. upsaliensis *strains, when a total of 17 *Campylobacter *strains (n = 4 *C. jejuni*; n = 2 *C. coli*; n = 1 *C. lari*; n = 2 *C. upsaliensis*; n = 2 *C. fetus*; n = 1 *C. concisus*; n = 1 *C. hyointestinalis*; n = 1 *C. mucosalis*; n = 3; *C. sputorum*) were examined [17]. In addition, three of seven *C. jejuni *isolates examined were found to carry fragmented 23S rRNA [18]. Moreover, the occurrence of fragmented 23S rRNA correlated with the presence of an IVS within the 23S rRNA genes. It was described that the presence of transcribed spacers is common in *Campylobacter *spp. (59%; n = 21 *C. jejuni *and n = 11 *C. coli*) [19]. All *Campylobacter *isolates containing transcribed spacers in their 23S rRNA gene sequences produced fragmented 23S rRNAs [19]. Most recently, among 104 strains of *C. coli *from turkeys, 69 strains harbored IVSs in all three 23S rRNA genes, whereas the other 35 strains lacked IVSs from at least one of the genes [20].

We have already reported the absence of IVSs shown in both the helix 25 (first quarter) and 45 (central) regions within 23S rRNA genes among a total of 65 isolates of *C. lari *[n = 38 urease-positive thermophilic *Campylobacter *(UPTC) [21] and n = 27 urease-negative (UN) *C. lari*] obtained from different sources and in several countries, by using PCR amplification, TA cloning and sequencing procedures [22]. In addition, the intact 23S rRNA was also identified in the *C. lari *isolates examined, resulting in no production of the fragmented 23S rRNA [22].

Thus, it would be important to clarify the molecular biological entities of the occurrence and the sequence structures of IVSs within the 23S rRNA genes in the much more isolates of several other species than *C. lari *of the genus *Campylobacter *including atypical species.

However, studies on molecular characterization and comparative analysis of IVSs within the 23S rRNA genes and these 23S rRNA fragmentations in much more than 200 *Campylobacter *isolates of *C. jejuni*, *C. coli*, *C. fetus*, and some other atypical *Campylobacter *species, namely *C. upsaliensis*, *C. hyointestinalis*, *C. sputorum *biovar sputorum, biovar fecalis, biovar paraureolyticus, *C. concisus *and *C. curvus *have not yet been reported. Therefore, we aimed to clarify molecular characteristics of IVSs within the 23S rRNA gene sequences and 23S rRNA fragmentations in these campylobacters other than *C. lari*, which has already been demonstrated not to harbor any IVSs [22]. In addition, the authors wished to comparatively analyze the IVSs among the *Campylobacter *organisms.

## Results

### IVSs in the helix 25 region

In the present study, two PCR primer pairs, f-/r-Cl23h25, designed to generate the helix 25 (first quarter) and, f-/r-Cl23h45, the helix 45 (central) regions within the 23S rRNA gene sequences with the 204 *Campylobacter *isolates were employed.

**Figure 1:**
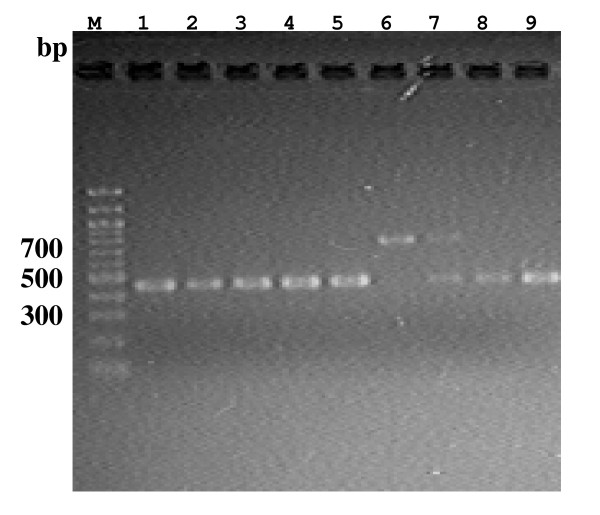
**Profiles of PCR products amplified with *Campylobacter *isolates using a primer pair of f-/r-Cl23h25**. Lane M, 100 bp DNA ladder (New England Biolabs Inc. England, UK); Lane 1, *C. jejuni *81-176; lane 2, *C. coli *165; lane 3, *C. upsaliensis *LMG8850; lane 4, *C. fetus *ATCC27374; lane 5, *C. hyointestinalis *ATCC35217; lane 6, *C. sputrum *bv. sputorum LMG7975; lane 7, *C. sputorum *bv. fecalis LMG8531; lane 8, *C. concisus *LMG7789; lane 9, *C. curvus *LMG7609.

**Figure 2:**
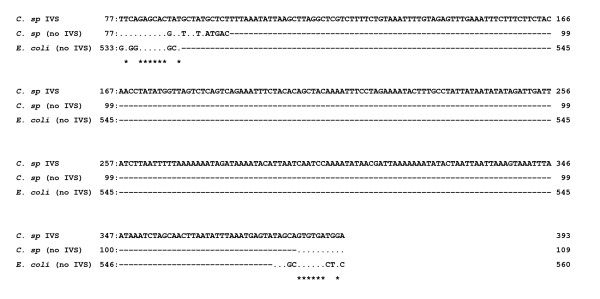
**Sequence alignment analysis in the helix 25 within 23S rRNA gene sequences from *Campylobacter *isolates**. Numbers at the left and right refer to the nucleotide positions determined in the present study. Dots indicate identical bases; changes are explicitly indicated: dashes are deletions; identical positions in all cases are marked by asterisks. Nucleotide sequence data in the helix 25 region within the *rrnB *operon from the *Escherichia coli *strain (J01695), identified to lack IVSs, were also aligned for comparison. *C. sp*, *C. sputorum*

When PCR was first carried out on the 204 isolates using the primer pair (f-/r-Cl23h25), amplicons were generated. Some of the examples are shown in Fig. 1. Following sequencing and analysis, only the four cases, *C. sputorum *biovar sputorum LMG7975 and biovar fecalis LMG8531, LMG8534 and LMG6728 isolates, were shown to carry IVSs in the helix 25 region among these isolates of more than 200. The sequence data in the helix 25 region from *C. sputorum *isolates are aligned in Fig. 2. As shown in Fig. 2, identical IVS occurred in the helix 25 region within 23S rRNA genes from the four *C. sputorum *isolates. Regarding the three *C. sputorum *biovar fecalis isolates, moreover, two different kinds of the 23S rRNA genes were identified to occur with and without the IVS, respectively (Fig. 2).

### IVSs in the helix 45 region

**Figure 3:**
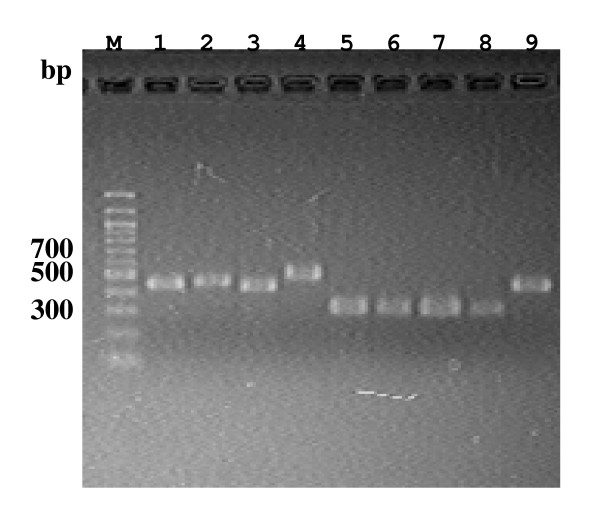
**Electrophoretic profiles of PCR products amplified with *Campylobacter *isolates using a primer pair of f-/r-Cl23h45**. For lane M and lane 1 to 9, see the legend to the Figure 1.

**Figure 4:**
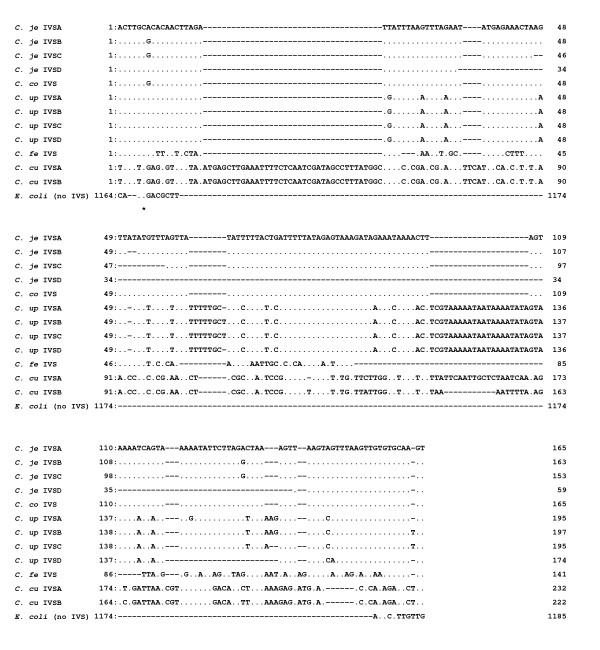
**Sequence alignment analysis in the helix 45 within 23S rRNA gene sequences from *Campylobacter *isolates**. *C. je*, *C. jejuni*;*C. co*, *C. coli*;*C. up*, *C. upsaliensis*;*C. fe*, *C. fetus*;*C. cu*, *C. curvus. C. je *IVSA, 86-375; B, 85-3; C, HP5090; D, HP5100; *C. co*, 27; *C. up *IVSA, G1104; B, 60-1; C, 2; D, 15; *C. fe*, cf2-1; *C. cu *IVSA, LMG7610; B, LMG11033.

Then, we carried out PCR amplification of the IVSs, in the central region (helix 45 region) within 23S rRNA gene sequences with the 204 *Campylobacter *isolates, using the primer pair f-/r-Cl23h45. Some examples of the PCR amplicons are shown in Fig. 3. Following sequencing and alignment analyses, in the helix 45 region, 30 C. hyointestinalis, fourteen *C. sputorum *biovar sputorum, biovar fecalis and paraureolyticus and 10 *C. concisus *isolates were shown not to carry any IVSs. In addition, however, regarding the other *Campylobacter *organisms examined in the present study, 30 of 56 *C. jejuni *(54%), 5 of 11 *C. coli *(45%), 25 of 33 *C. fetus *(76%), 30 of 43 *C. upsaliensis *(70%) and 6 of 7 *C. curvus *(86%) isolates were shown to carry the IVSs in the helix 45 region. Some of the sequence data of the IVSs in the helix 45 region were aligned in Fig. 4. Regarding the IVS sequences in the helix 45 region, four IVSs with similar sequences occurred in the *C. jejuni *and *C. upsaliensis *species, respectively, and two also in the *C. curvus *isolates (Fig. 4 and Table 1). In addition, one kind of IVS with an identical sequence occurred in the *C. coli *and *C. fetus *isolates, respectively (Fig. 4), Moreover, the eight IVSs in the *C. jejuni *and *C. upsaliensis *isolates showed high sequence similarities to each other (~90%), and one kind of IVS in the *C. jejuni *and *C. coli *showed an identical sequence (Fig. 4). Four kinds of IVSs in the *C. upsaliensis *isolates, interestingly, carried two characteristic insertion sequences of several base pairs (bp) and twenty and several bp at the two positions (Fig. 4). In *C. jejuni *(isolates nos. HP5075 and HP5095) and *C. upsaliensis *(26-4 and 40-1), two different kinds of the 23S rRNA genes were identified to occur with and without the IVS in the helix 45 region. Moreover, multiple and heterogeneous IVSs were shown in *C. upsaliensis *48-1 and 68-3 isolates, respectively. Consequently, identification of the IVSs within the 23S rRNA genes from the 207 *Campylobacter *isolates is summarized in the Table 1.

**Table 1:** IVSs within 23S rRNA genes from *Campylobacter *organisms analyzed in the present study

Organism	Isolate	IVS name	Accession No.
*C. sputorum*	LMG7975	*C. sp *IVS	AB491949
*C. sputorum*	LMG8535	*C. sp *no IVS	AB491950
*C. jejuni*	86-375	*C. je *IVSA	AB491951
*C. jejuni*	85-3	*C. je *IVSB	AB491952
*C. jejuni*	HP5090	*C. je *IVSC	AB491953
*C. jejuni*	HP5100	*C. je *IVSD	AB491954
*C. coli*	27	*C. co *IVS	AB491955
*C. upsaliensis*	G1104	*C. up *IVSA	AB491956
*C. upsaliensis*	60-1	*C. up *IVSB	AB491957
*C. upsaliensis*	2	*C. up *IVSC	AB491958
*C. upsaliensis*	15	*C. up *IVSD	AB491959
*C. fetus*	cf2-1	*C. fe *IVS	AB491960
*C. curvus*	LMG7610	*C. cu *IVSA	AB491961
*C. curvus*	LMG11033	*C. cu *IVSB	AB491962

### Secondary structure models of the IVSs

**Figure 5:**
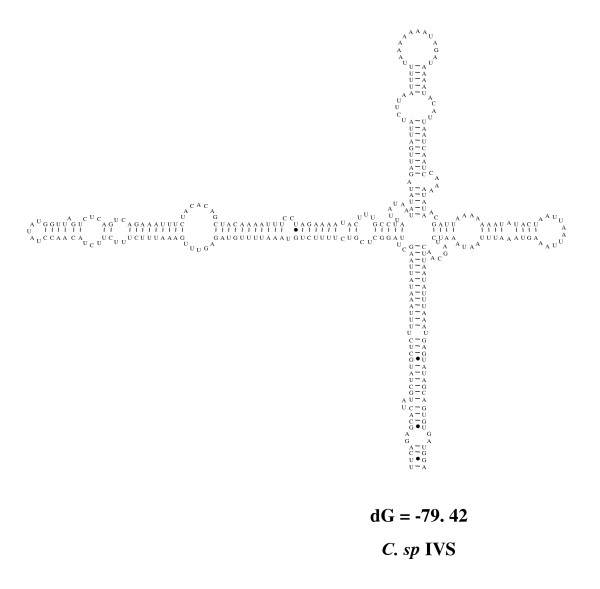
**Secondary structures of IVSs in the helix 25 region from *C. sputorum *biovar sputorum LMG7975**. Some details of the IVSs were shown in Table 1. Secondary structure predictions were obtained using the mfold server available at bioinfo's home page.

**Figure 6:**
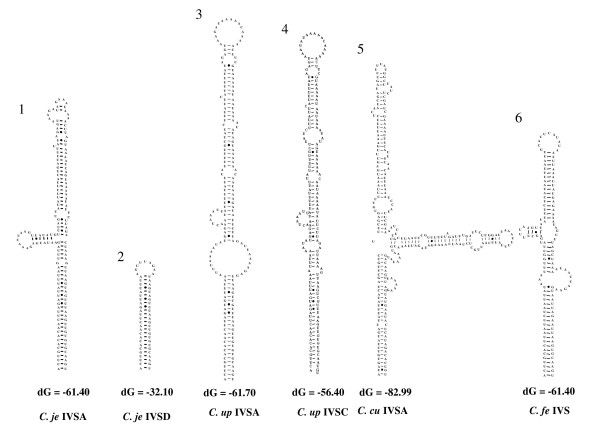
**Secondary structures of IVSs in the helix 45 region from *Campylobacter *isolates**. For other details, refer to legend to Figure 4.

Regarding the IVSs identified in the present study, within the 23S rRNA gene sequences from the *Campylobacter *isolates examined, secondary structure models were constructed with all the IVSs shown in Table 1. Fig. 5 and 6 show some examples of the secondary structure models of the IVSs in helix 25 (the first quarter; Fig. 5) and helix 45 (central; Fig. 6) regions. In the present models, stem and loop structures were identified in all IVSs.

### Gel electrophoresis of purified RNA

**Figure 7:**
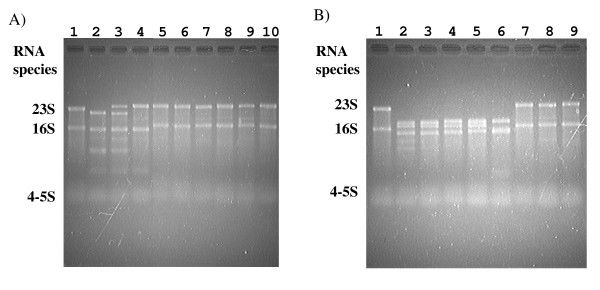
**Electrophoretic profiles of purified RNA from the *Campylobacter *isolates containing IVSs**. In the helix 25 (A) and 45 (B) regions within 23S rRNA genes. Purified RNA from *E. coli *DH5α was employed as a reference marker (lane 1). (A) Lane 2, *C. sputorum *bv. sputorum LMG7975; lane 3, bv. fecalis LMG 8531; lane 4, bv. fecalis LMG 11761; lane 5, *C. coli *NCTC11366; lane 6, *C. upsaliensis *12-1; lane 7, *C. fetus *8414c; lane 8, *C. hyointestinalis *ATCC35217; lane 9, *C. concisus *LMG 7789; lane 10, *C. curvus *LMG13935. (B) Lane 2, *C. jejuni *81-176; lane 3, *C. coli *165; lane 4, *C. upsaliensis *LMG8850; lane 5, *C. fetus *ATCC27374; lane 6, *C. curvus *LMG 7609; lane 7, *C. upsaliensis *12-1; lane 8, *C. fetus *8414c; lane 9. *C. hyointestinalis *ATCC35217.

Denaturing agarose gel electrophoresis profiles of purified RNA from the *Campylobacter *isolates was carried out to clarify if the primary RNA transcripts of 23S rRNA were fragmented in the isolates or not. Purified RNA from *E. coli *DH5α cells, identified to lack IVSs, was also employed as a reference marker (lane 1 in Fig. 7). In the purified RNA fraction from the isolates of *C. sputorum *biovar sputorum LMG7975 (lane 2), whose 23S rRNA gene(s) was demonstrated to carry IVSs in the helix 25, no 23S rRNA was evident in the fraction (Fig. 7A). Instead of the 23S rRNA, other smaller RNA fragments were identified (lane 2 in Fig. 7A). Regarding the *C. sputorum *biovar fecalis LMG8531, two large rRNA bands consisting of an intact and a fragmented 23S rRNAs, were identified to occur in the isolate (lane 3). Some other examples of 23S rRNAs whose genes were identified not to carry IVSs in the helix 25 region, are also shown in the Figure. (lanes 4, 5, 6, 8, 9 and 10 in Fig. 7A). Thus, intact 23S rRNAs were identified in *Campylobacter *isolates containing no IVSs in the helix 25 region. In addition, in Fig. 7B, some of the denaturing agarose gel electrophoresis profiles of purified RNA from the *Campylobacter *isolates, whose helix 45 regions were examined, are shown. No 23S rRNA and fragmented other smaller RNA fragments were evident in the some purified RNA fractions, and intact 23S rRNAs were evident in other RNA fractions.

In relation to the 16S rRNA molecules from the four isolates of *C. sputorum *biovar sputorum LMG7975 (lane 2), biovar fecalis LMG8531 (lane 3) and LMG11763 (lane 4 in Fig. 7A) and *C. curvus *LMG7609 (lane 6 in Fig. 7B), surprisingly, slightly shorter RNAs than the 16S were identified in these isolates, instead of the 16S rRNA species.

## Discussion

We have already shown no IVSs, in the helix 25 regions within the 23S rRNA genes among a total of 65 isolates of *C. lari *[n = 27 UN *C. lari*; n = 38 UPTC [22]. Consequently, in 265 isolates of 269 *Campylobacter *isolates of the nine species (n = 56 *C. jejuni*; n = 11 *C. coli*; n = 33 *C. fetus*: n = 65 *C. lari*; n = 43 *C. upsaliensis*; n = 30 *C. hyointestinalis*; n = 14 *C. sputorum*; n = 10 *C. concisus*; n = 7 *C. curvus*) examined, the absence of IVSs was identified in helix 25 region within 23S rRNA genes. Moreover, until now, no IVSs have been identified in the helix 25 region within 23S rRNA genes, from more than 100 *Campylobacter *isolates of the 8 species (*C. jejuni*, *C. fetus*, *C. upsaliensis*, *C. coli*, *C. lari*, *C. concisus*, *C. hyointestinalis*, *C. mucosalis*) by other research groups [17-20]. Thus, IVS is extremely rare in the helix 25 region within the 23S rRNA genes from the *Campylobacter *organisms. Therefore, this is the first scientifically significant report of IVSs in the helix 25 from *C. sputorum *biovar sputorum and biovar fecalis among *Campylobacter *organisms. In addition, no IVSs have been identified to occur in the helix 45 from *C. sputorum *strains (*C. sputorum *biovar bubulus, biovar fecalis and biovar sputorum) [17]. Regarding the 23S rRNA, however, fragments smaller than intact 23S rRNA were visible on the gel for *C. sputorum *biovar bubulus and fecalis strains by using a northern blot hybridization analysis [17].

In relation to the IVSs in the helix 45 from the *C. jejuni *and *C. coli *isolates, a total of 149 isolates (n = 32 *C. jejuni*; n = 117 *C. coli*) have already been examined [17-20]. In the two major and typical *C. jejuni *and *C. coli *species of *Campylobacter*, IVSs occur in helix 45 at high percent degree (59% for *C. jejuni *n = 32; 84% for *C. coli *n = 117) [2,6,19,20]. In the present study, the occurrence of IVSs with the two typical *Campylobacter *species, were shown in helix 45 region at a high similar percentage (54% for *C. jejeuni *n = 56; 45% for *C. coli *n = 11), as shown in Table 2. In addition, IVSs have already been shown to occur in the helix 45 region for only a few other *Campylobacter *species, than the typical *C. jejuni *and *C. coli *(n = 2 *C. upsaliensis*; n = 2 *C. fetus*; n = 1 *C. concisus*; n = 1 *C. hyointestinalis*; n = 1 *C. mucosalis*; n = 3 *C. sputorum*), three IVSs being identified to occur in *C. fetus *and in *C. upsaliensis *[17]. At present, we identified the majority (62/83) of isolates from the three *Campylobacter *species of *C. fetus*, *C. upsaliensis *and *C. curvus *to carry IVSs in helix 45 within 23S rRNA genes. However, in a total of 54 isolates of the three *Campylobacter *species of *C. hyointestinalis *(n = 30), *C. sputorum *(n = 14) and *C. concisus *(n = 10), no IVSs were identified in helix 45 region, as shown in Table 2. These are also scientifically significant observations. Thus, in conclusion, no IVSs were identified in 105 isolates of three *Campylobacter *species (*C. hyointestinalis*, *C. concisus *and *C. lari*) both in the 25 and 45 helix regions within the 23S rRNA genes.

**Table 2:** Summary of identification of IVSs within 23S rRNA genes from *Campylobacter *organisms analyzed in the presen study

*Campylobacter *species	IVS in helix 25	IVS in helix 45
*C. jejuni *(n = 56)	0	30
*C. coli *(n = 11)	0	5
*C. fetus *(n = 33)	0	25
*C. upsaliensis *(n = 43)	0	30
*C. hyointestinalis *(n = 30)	0	0
*C. sputorum *biovar sputorum (n = 4)	1	0
*C. sputorum *biovar fecalis (n = 5)	3	0
*C. sputorum *biovar paraureolyticus (n = 5)	0	0
*C. concisus *(n = 10)	0	0
*C. curvus *(n = 7)	0	6
*C. lari *(n = 65)	0	0

Total (n = 269)	4	96

Overall, in the present study, two different kinds of the 23S rRNA genes with and without the IVSs occurred in the seven *Campylobacter *isolates (n = 3 *C. sputorum *biovar fecalis; n = 2 *C. jejuni*; n = 2 *C. upsaliensis*) (data not shown).

In addition, in the present study, electrophoretic profiles of the purified RNA from *Campylobacter *organisms were examined. In the purified RNA fractions of some isolates from *C. sputorum *and *C. curvus*, slightly shorter 16S rRNA were identified to occur (lanes 2, 3 and 4 in Fig. 7A and lane 6 in Fig. 7B), as described above. These may be partially due to occurrence of IVS within the 16S rRNA genes from these isolates and fragmentation of the primary 16S rRNA transcripts among these isolates. However, we have not clarified the nature of the 16S rRNA genes from these isolates, yet. Therefore, sequencing and alignment analyses of the complete 16S rRNA genes from these isolates are needed to identify the nature of the rRNA from these two *Campylobacter *species. Research to examine this is now in progress.

## Conclusions

Consequently, in 267 isolates of 269 *Campylobacter *isolates of the nine species (n = 56 *C. jejuni*; n = 11 *C. coli*; n = 33 *C. fetus*: n = 65 *C. lari*; n = 43 *C. upsaliensis*; n = 30 *C. hyointestinalis*; n = 14 *C. sputorum*; n = 10 *C. concisus*; n = 7 *C. curvus*) examined, the absence of IVSs was identified in helix 25 region within 23S rRNA genes. Thus, IVS is extremely rare in the helix 25 region within the 23S rRNA genes from the *Campylobacter *organisms.

The occurrence of IVSs with the two typical *Campylobacter *species, were shown in helix 45 region at a high percentage (54% for *C. jejeuni *n = 56; 45% for *C. coli *n = 11). We also identified the majority (62/83) of isolates from the three *Campylobacter *species of *C. fetus*, *C. upsaliensis *and *C. curvus *to carry IVSs in helix 45. However, in a total of 54 isolates of the three species of *C. hyointestinalis *(n = 30), *C. sputorum *(n = 14) and *C. concisus *(n = 10), no IVSs were identified in the region.

Thus, in conclusion, no IVSs were identified in 105 isolates of three *Campylobacter *species (*C. hyointestinalis*, *C. concisus *and *C. lari*) both in the 25 and 45 helix regions. In addition, intact 23S rRNAs were identified in the purified RNA fractions in *Campylobacter *isolates containing no IVSs, and no 23S rRNA and fragmented other smaller RNA fragments were evident in the isolates containing IVSs.

## Methods

### *Campylobacter *isolates and genomic DNA preparation

A total of 204 *Campylobacter *isolates [*C. jejuni *(n = 56); *C. coli *(n = 11); *C. fetus *(n = 33) *C. upsaliensis *(n = 43); *C. hyointestinalis *(n = 30); *C. sputorum *biovar sputorum (n = 4); biovar fecalis (n = 5); biovar paraureolyticus (n = 5); *C. concisus *(n = 10); *C. curvus *(n = 7)] were used in the present study (Table 2). Genomic DNA was prepared from *Campylobacter *cells by cethyltrimethyl ammonium bromide and proteinase K treatments, phenol-chloroform extraction and ethanol precipitation [23].

### PCR amplification, cloning and sequencing

**Figure 8:**
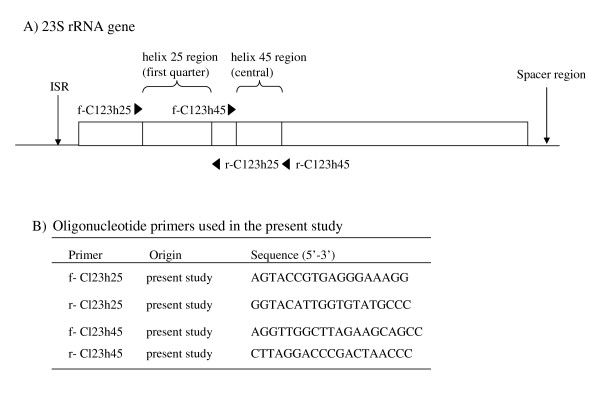
**A schematic representation of a 23S rRNA gene including two helix regions**. Arrows indicate primer site for PCR amplification (A). Sequences of oligonucleotide primers used in the present study (B). ISR, internal spacer region.

We have already designed two PCR primer pairs, f-/r-Cl23h25, constructed to amplify helix 25 region and f-/r-Cl23h45, helix 45 region within the 23S rRNA gene sequences, based on the 23S rRNA gene sequence information from 12 UPTC isolates (DDBJ/EMBL/GenBank accsssion numbers, AB287301-AB287312), *C. jejuni *TGH9011 (Z29326) and *C. coli *VC167 (U09611) (Fig. 8) [22].

PCR products, separated by 1% (w/v) agarose gel electrophoresis in 0.5× TBE, were purified with QIAquick PCR Purification Kit (QIAGEN, Tokyo, Japan). The purified amplicons were subjected to cycle sequencing with BigDye Terminator (Applied Biosystems, Tokyo, Japan) and with the PCR primers (f-/r-Cl23h25 or f-/r-Cl23h45) and the reaction products were separated and detected with an ABI PRIM™ 3100 Genetic analyzer (Applied Biosystems). When any multiple IVSs were suggested to occur from the cycle sequencing profiles, the purified amplicons were then cloned into pGEM-T vector (Promega Corp. Tokyo, Japan) and the ligated recombinant DNA was transformed into competent *Escherichia coli *JM109 cells, [23]. Following the nucleotide sequencing reaction with M13, sequencing of the amplicons was performed with Hitachi SQ5500EL DNA autosequencer (Hitachi Electronics Engineering Co., Tokyo, Japan).

### Nucleotide sequence analysis

Nucleotide sequence analysis was carried out by using the GENETYX-Windows computer software (version 9; GENETYX Co., Tokyo, Japan). Nucleotide sequences of the helix 25 and 45 regions within the 23S rRNA gene sequences from the isolates of campylobacters were compared to each other and with the accessible sequence data from other campylobacters using CLUSTAL W software, respectively (1.7 program) [24], which was incorporated in the DDBJ/EMBL/GenBank databases. The sequence data of the IVSs determined in the present study are accessible in the DDBJ/EMBL/GenBank under accession numbers shown in Table 1.

### Secondary structure predictions

Secondary structure predictions of the IVSs in the helix 25 and 45 within 23S rRNA genes from *Campylobacter *isolates were obtained by using the mfold server available at bioinfo's home page http://www.bioinfo.rpi.edu/applications/mfold/rna/forml.cgi.

### Total cellular RNA extraction and RNA gel electrophoresis

Total cellular RNA was extracted and purified from *Campylobacter *cells by using RNAprotect Bacteria Reagent and RNeasy Mini Kit (QIAGEN). RNAs were analyzed by denaturing 1% (w/v) agarose gel electrophoresis in 1% (w/v) MOPS (3-morpholinopropanesulfonic acid) containing 2% (w/v) formaldehyde after heat denaturation of the total RNA at 65°C for 15 min. RNAs were visualized by ethidium bromide staining.

## List of abbreviations used

*C*: *Campylobacter*; IVS: intervening sequence; rRNA: ribosomal RNA; *E. coli*: *Escherichia coli*.

## Authors' contributions

MM participated in design of the study, collected strains, drafted the manuscript and review of the manuscript. AT, and YK were involved with cloning, sequencing and analysis of the rRNA gene sequences from *Campylobacter *strains. NM also collected strains. JEM and BCM participated in its design and coordination, and review of the manuscript. All authors have read and approved the final version of this paper.
